# Roles of Adipokines in Digestive Diseases: Markers of Inflammation, Metabolic Alteration and Disease Progression

**DOI:** 10.3390/ijms21218308

**Published:** 2020-11-05

**Authors:** Ming-Ling Chang, Zinger Yang, Sien-Sing Yang

**Affiliations:** 1 Department of Medicine, College of Medicine, Chang Gung University, Taoyuan 33305, Taiwan; 2 Division of Hepatology, Department of Gastroenterology and Hepatology, Chang Gung Memorial Hospital, Taoyuan 33305, Taiwan; 3 Program in Molecular Medicine, University of Massachusetts Medical School, Worcester, MA 01655, USA; zingery@gmail.com; 4 Liver Center, Cathay General Hospital Medical Center, Taipei 10630, Taiwan; yangsien@hotmail.com

**Keywords:** adipokine, leptin, adiponectin, NAFLD, HBV, HCV, pancreas, esophagus, stomach, colon, small intestine, biliary, gallbladder

## Abstract

Adipose tissue is a highly dynamic endocrine tissue and constitutes a central node in the interorgan crosstalk network through adipokines, which cause pleiotropic effects, including the modulation of angiogenesis, metabolism, and inflammation. Specifically, digestive cancers grow anatomically near adipose tissue. During their interaction with cancer cells, adipocytes are reprogrammed into cancer-associated adipocytes and secrete adipokines to affect tumor cells. Moreover, the liver is the central metabolic hub. Adipose tissue and the liver cooperatively regulate whole-body energy homeostasis via adipokines. Obesity, the excessive accumulation of adipose tissue due to hyperplasia and hypertrophy, is currently considered a global epidemic and is related to low-grade systemic inflammation characterized by altered adipokine regulation. Obesity-related digestive diseases, including gastroesophageal reflux disease, Barrett’s esophagus, esophageal cancer, colon polyps and cancer, non-alcoholic fatty liver disease, viral hepatitis-related diseases, cholelithiasis, gallbladder cancer, cholangiocarcinoma, pancreatic cancer, and diabetes, might cause specific alterations in adipokine profiles. These patterns and associated bases potentially contribute to the identification of prognostic biomarkers and therapeutic approaches for the associated digestive diseases. This review highlights important findings about altered adipokine profiles relevant to digestive diseases, including hepatic, pancreatic, gastrointestinal, and biliary tract diseases, with a perspective on clinical implications and mechanistic explorations.

## 1. Introduction

**Figure 1:**
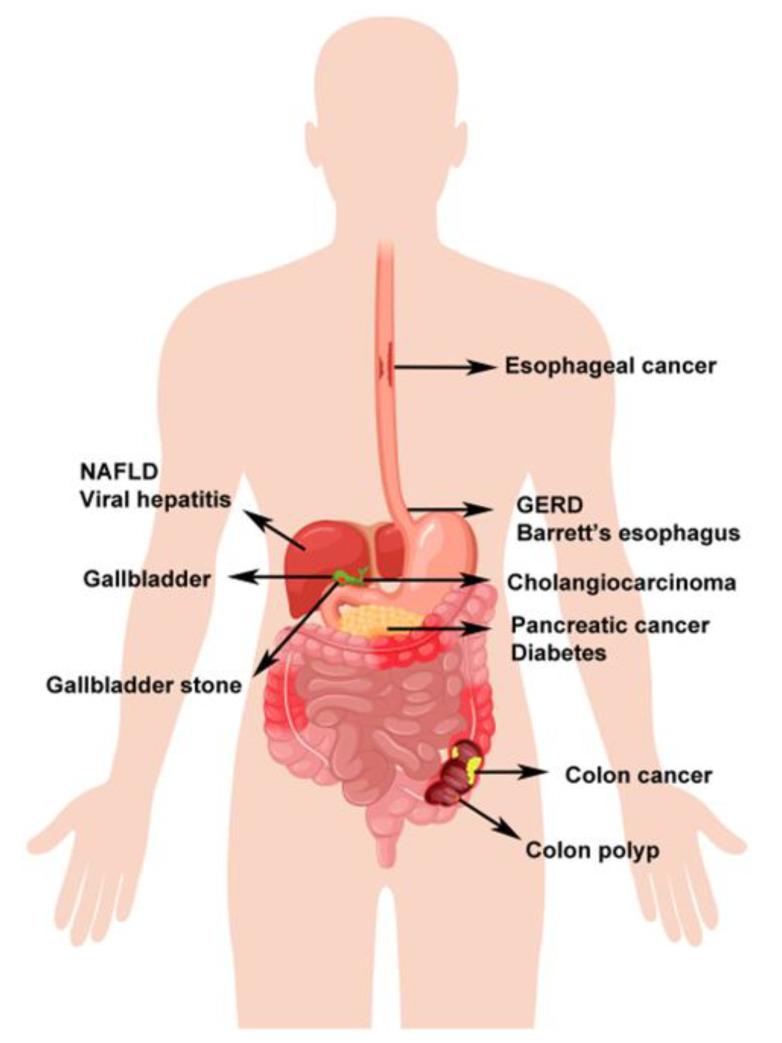
A schematic describing obesity-related digestive diseases. The obesity-related diseases in the digestive tract from the esophagus, stomach, liver, biliary tree, gallbladder to the colon are labelled. Ca: cancer. GERD: gastroesophageal reflux disease; NAFLD: non-alcoholic fatty liver disease.

Adipose tissue is recognized as a highly dynamic endocrine tissue exhibiting extensive physiological functions [1] and is composed of mature adipocytes and a stromal vascular fraction, where adipose-derived stem cells, blood cells, fibroblasts, and nerves reside [2]. Adipose tissue constitutes a central node in the interorgan crosstalk network and mediates the regulation of multiple organs and tissues through adipokines [3] (also called adipocytokines), biologically active molecules causing pleiotropic effects, including modulation of angiogenesis, metabolism, and inflammation [4]. The emerging functional characterization of adipokines suggests a close link between the endocrine and immune systems of adipose tissue. This link is emphasized by the altered expression pattern of adipokines in adipose tissue adjacent to sites of inflammation [5]. Obesity, the excessive accumulation of adipose tissue due to hyperplasia and hypertrophy [6], is currently considered a global epidemicand is related to low-grade systemic inflammation. This state of inflammation is characterized by alterations in adipokine regulation [7]. Interestingly, digestive cancers such as gastric and colon cancers grow anatomically near adipose tissue. During their interaction with cancer cells, adipocytes dedifferentiate into preadipocytes or are reprogrammed into cancer-associated adipocytes, which secrete adipokines to stimulate the adhesion, migration, and invasion of tumor cells [8]. In particular, the liver is the central metabolic hub for carbohydrate, lipid, and protein metabolism [9]. Adipose tissue and the liver play important roles in the regulation of whole-body energy homeostasis, and prolonged metabolic stress leads to adipose tissue dysfunction, inflammation, and adipokine release, causing increased lipid flux to the liver, resulting in fatty liver [10]. Moreover, adipokines are involved in modulating insulin resistance, which is at the heart of obesity-related digestive diseases [11], including gastroesophageal reflux disease (GERD), Barrett’s esophagus, esophageal cancer, colon polyps and cancer, non-alcoholic fatty liver disease (NAFLD), viral hepatitis, cholelithiasis, gallbladder cancer, cholangiocarcinoma, pancreatic cancer, and diabetes [12,13] (Figure 1). 

However, whether dysregulated adipokines are merely the consequence of digestive disease or whether these altered adipokines promote disease progression is unknown, and the roles of adipokines in digestive diseases remain to be investigated.

Leptin was the first adipokine to be discovered in 1994 [14], and hundreds of adipokines have since been discovered [15]. For example, adiponectin is an anti-inflammatory and insulin-sensitizing adipokine and is secreted mainly by white adipose tissue; however, adiponectin is decreased in obesity [16], and low serum adiponectin is associated with many cancers and inflammatory diseases, such as colon cancer and colitis [17]. The specific alteration patterns of adipokine expression and the associated basis in the development of various digestive diseases might contribute to the identification of prognostic biomarkers as well as therapeutic and preventative approaches for the associated diseases. The current review thus systematically highlights important findings about altered adipokine profiles in the context of diseases of the digestive tract, including the liver, pancreas, esophagus, stomach, small intestine, and colon, with a perspective on the clinical implications and associated mechanistic approaches.

## 2. Adipokines and the Liver

### 2.1. NAFLD

NAFLD, characterized by the accumulation of fat in the liver [18] due to non-alcoholic causes, is the liver manifestation of metabolic syndrome and includes the spectrum of hepatic steatosis and non-alcoholic steatohepatitis (NASH) [19]. Systemic insulin resistance is a major driver of hepatic steatosis in NAFLD, while lipotoxicity of accumulated lipids along with activation of the innate immune system are major drivers of NASH [18]. Thus, many adipokines have evolved as crucial signals in NAFLD.

#### 2.1.1. Leptin

Increased leptin levels act as a pro-inflammatory stimulus [20], and leptin increases susceptibility to hepatotoxicity by regulating cytokine production and T cell activation [21]. On the other hand, leptin augments the oxidation of fatty acids in the liver by activating peroxisome proliferator-activated receptor-alpha (PPAR-α) [22]. Higher levels of circulating leptin were found to be associated with increased severity of NAFLD [23]. Moreover, polymorphisms in the leptin receptor (ObR) gene have been reported to be related to NAFLD [24]. However, in contrast to patients with obesity-associated NAFLD, patients with lipodystrophy have low levels of adipokines, including leptin [25], and leptin therapy thus appears to be highly effective for NASH in hypoleptinemic lipodystrophic patients [26].

#### 2.1.2. Adiponectin

Adiponectin enhances glucose and fatty acid oxidation, improves insulin sensitivity, attenuates plaque formation, and increases aldosterone production [27]. The hepatoprotective effects of adiponectin, including its antisteatotic, anti-inflammatory, and antifibrogenic effects, have been widely investigated. Adiponectin levels are reduced in individuals with NAFLD [28] and are inversely related to the severity of steatosis, necroinflammation [29], and fibrosis [28]. Hypoadiponectinemia may play an important pathophysiological role in the progression from non-alcoholic fatty liver to NASH [30]. The adiponectin signaling pathway in the liver acts through T-cadherin, adiponectin receptor 1 (AdipoR1), AdipoR2, AMP-activated protein kinase (AMPK), ceramidase activity, and an adaptor protein, phosphotyrosine interacting with a PH domain and leucine zipper 1, and the recently discovered suppressor of glucose from autophagy [31]. AdipoR1 is expressed abundantly in muscle, whereas AdipoR2 is predominantly expressed in the liver [32]. NAFLD is associated with decreased hepatic expression of the two adiponectin receptors (AdipoR1 and 2), thereby contributing to a state of hepatic adiponectin resistance [33]. Comprehensive crosstalk between adiponectin and its cognate receptors, specifically AdipoR2, in the liver attenuates hepatic lipoinflammation by interacting with hepatic PPARs [34]. In addition, adiponectin protects hepatocytes from tumor necrosis factor-alpha (TNF-α)-induced death [35]; specifically, adiponectin is a potent TNF-α-neutralizing adipokine [36]. Moreover, bile acid (BA) synthesis and serum BA levels are directly correlated with disease severity in NAFLD, while the adiponectin level is inversely correlated with this parameter [37]. Furthermore, the single-nucleotide polymorphism (SNP) rs1501299 in the adiponectin gene might be related to increased NAFLD susceptibility [38].

#### 2.1.3. Other Adipokines

Interestingly, our previous case-control study showed that plasminogen activator inhibitor-1 (PAI-1) is independently associated with NAFLD after adjustment for leptin and adiponectin levels [39]. However, data regarding other adipokines, including resistin (RETN), visfatin (i.e., extracellular nicotinamide phosphoribosyltransferase (eNAMPT)), retinol-binding protein-4 (RBP-4), chemerin, adipsin, obestatin, omentin, and vaspin, in NAFLD are inconclusive or limited [40].

### 2.2. Viral Hepatitis

#### 2.2.1. Hepatitis B

Chronic hepatitis B virus (HBV) infection represents a major global health issue, affecting an estimated 257–291 million persons worldwide and is associated with substantial morbidity and mortality because of complications, including hepatitis, cirrhosis, and hepatocellular carcinoma (HCC) [41]. HBV is a hepatotropic, noncytopathic member of the hepadnaviridaefamily, comprising a 3.2 kb partly double-stranded, relaxed circular DNA genome and viral DNA polymerase condensed into a nucleocapsid by hepatitis B core proteins. There are now known to be at least tengenotypes of HBV [42]. Current therapies for chronic hepatitis B (CHB) remain limited to pegylated-interferon-alpha (PegIFN-α), or any of the fiveapproved nucleos(t)ide analog (Nuc) treatments. While viral suppression can be achieved in the majority of patients with the high-barrier-to-resistance new-generation of Nuc, HBsAg loss is achieved by PegIFN-α and/or Nuc in only 10% of patients after a 5-year follow-up [43].

##### Leptin

Leptin levels may be related to fibrosis progression in nondiabetic patients with chronic HBV infection [44]. Consistent with this possibility, cirrhosis due to CHB is associated with high leptin levels, which constitute a negative prognostic factor for the response to lamivudine monotherapy in patients with CHB [45]. Additionally, increased baseline leptin levels were noted in CHB patients compared to controls, and leptin levels decreased during IFN-α treatment [46]. However, decreased serum leptin levels were ever found in patients with HBV-related cirrhosis and HCC [47].

##### Adiponectin

In HepG2-HBV-stable cells, HBV replication was found to be upregulated by adiponectin and downregulated by adiponectin-targeting small interfering RNAs [48]. Consistent with this finding, individuals with chronic HBV infection have high serum adiponectin levels. Particularly in overweight and obese HBV-infected patients, a high HBV load was found to be positively associated with serum adiponectin levels [49]. Intriguingly, in HBV-infected male subjects without diabetes, the serum HBV DNA level correlated inversely with the serum high-density lipoprotein cholesterol level, and patients with detectable HBV DNA had lower adiponectin levels than those without [50]. Regarding hepatic inflammation, alanine aminotransferase (ALT) levels were found to be inversely related to adiponectin levels, independent of metabolic factors and HBV status [51], andadiponectin levels tended to decrease in HBV responders following IFN-α therapy [52]. On the other hand, adiponectin levels were associated with an increased risk of HCC in HBV patients. Over time, participants with higher adiponectin levels were less likely to achieve seroclearance of HBV surface antigen (HBsAg) and more likely to have persistently higher HBV DNA levels. Eventually, they were also more likely to develop cirrhosis [53].

##### Resistin

HBV-infected patients were found to show increased levels of serum resistin, and high serum resistin levels were associated with intrahepatic inflammation and necrosis [54]. In addition, resistin levels decreased in HBV-infected patients after antiviral therapy, especially in the subgroup of responders [55].

##### Visfatin

Visfatin concentrations were found to be elevated and negatively correlated with haptoglobin and fibrinogen levels in patients with chronic HBV infection [56].

##### Chemerin

Although chemerin is protective in experimental models of HCC, chemerin was reported to be induced in tumor tissues of patients with HBV-related HCC [57].

##### Multiple Adipokines

CHB patients were found to have higher serum adiponectin and visfatin levels but lower leptin levels than healthy controls. Moreover, serum leptin, adiponectin, and visfatin levels were correlated with HBV viremia, HBsAg levels, and liver fibrosis stage [58].

#### 2.2.2. Hepatitis C

Hepatitis C virus (HCV), a human pathogen responsible for acute and chronic liver disease, has variants classified into eight genotypes [59] and chronically infects an estimated 71.1 million individuals worldwide [60]. HCV is currently thought to cause metabolic alterations in addition to a simple hepatic viral infection, as it affects insulin signaling, and much of its life cycle is closely associated with lipid metabolism [61]. Because both HCV infection and alterations in adipokines are critical in metabolism, their potential relationship has attracted attention [62]. There are genotype-specific impacts on HCV-associated metabolic alterations [61]. The combination of PegIFN and ribavirin provided a “cure” for a considerable proportion of patients with HCV infection, particularly those with the favorable IFN λ3 (IFNL3) genotype [63]. These cure rates were further improved by replacing IFN-based therapy with potent direct-acting antiviral agents (DAAs) [64]. Thus, some cross-sectional studies, as well as many longitudinal studies of HCV-infected patients receiving IFN-based or DAA therapy, have provided a landscape in which to study metabolic alterations and the associated effects of HCV clearance by comparing adipokine profiles before and after anti-HCV treatment.

##### Leptin

In cross-sectional studies, increased [65,66] or unchanged [67,68] serum leptin levels in patients with chronic HCV infection compared with controls have been noted. Regarding genotype-specific characteristics, the connection between steatosis and leptin in patients infected with genotype (G) 1 or G2 HCV [69,70] has been reported. In addition, high baseline leptin levels have been reported to be negative predictors of a sustained virologic response (SVR) to IFN-based therapy [71,72]. Moreover, leptin levels were found to remain unchanged after IFN therapy in patients with chronic HCV infection who achieved SVRs; leptin and complement component 3 (C3) may maintain immune and metabolic homeostasis through association with C4 and total cholesterol [73].

##### Adiponectin

Increased adiponectin levels were noted in HCV-infected patients [74,75,76,77], especially those with severe fibrosis [78], compared with controls, suggesting a pattern of adiponectin resistance [67,76], although one study found similar adiponectin levels between HCV-infected patients and controls [67]. Studies involving various HCV genotypes have reported diverse findings regarding adiponectin alteration and its correlation with HCV viral load or disease progression. In cross-sectional studies, patients with G3 HCV infection were found to have lower adiponectin levels than patients infected with other genotypes of HCV [79]. High viral load and G2 HCV infection were found to be associated with low serum adiponectin levels [80], and adiponectin levels were found to increase with the progression of hepatic fibrosis but were not related to viral load in patients with G4 HCV infection [81]. In patients with G1 or G3 HCV infection, adiponectin levels were found to be linked with steatosis only in males and to increase with hepatic inflammation [82]. In addition, insulin resistance was found to be associated with a decrease in adiponectin levels in G3 HCV-infected patients but not in G1 HCV-infected patients [83]. However, adiponectin levels were found to be decreased in both G1 and G3 HCV-infected patients [84]. The lack of clarity regarding HCV infection and adiponectin alterations seems to stem from the heterogeneous hepatic pathologies, metabolic conditions, and immune reactions of the patients involved in various studies. In HCV-infected patients, hepatic fibrosis [76,81]/inflammation [85] and steatosis [79,84,86,87,88,89] are associated with hyperadiponectinemia and hypoadiponectinemia, respectively. Additionally, adiponectin was found to be negatively correlated with insulin resistance, hepatic steatosis, and metabolic syndrome [90]. Consistent with this finding, our previous study showed that HCV core-induced nonobese hepatic steatosis is associated with hypoadiponectinemia; however, these effects may be ameliorated by adiponectin treatment [91]. Moreover, an anti-HCV-specific immune response was found to be strongly associated with higher serum total adiponectin and high-molecular-weight (HMW) adiponectin levels [84]. Whether HCV viral clearance leads to hyper or hypoadiponectinemia remains unclear and may differ between G3 and G4 HCV infections [81,92]. However, a large cohort study of 747 consecutive patients with G1, G2, G3, and G6 HCV infection showed that the adiponectin level and the aminotransferase-to-platelet ratio index decreased 24 weeks post-therapy in patients with SVR. During HCV infection, adiponectin may affect insulin sensitivity through triglycerides. After viral clearance, adiponectin levels decrease; moreover, they are directly associated with insulin sensitivity and decrease upon the improvement of hepatic fibrosis [93]. Thus, after SVR, the decrease in adiponectin in G4 HCV-infected patients [81] may reflect the reversal of hepatic fibrosis and hypotriglyceridemia, whereas the increase in adiponectin in G3 HCV-infected patients [92] may indicate an improvement in hepatic steatosis, which is most evident in G3 HCV-infected patients [94]. Hepatic steatosis associated with infection with G3 but not other genotypes of HCV was improved after SVR [95]. Regarding HCC, in G1 HCV-associated HCC, baseline adiponectin levels were found to be positively associated with the occurrence of HCC, independent of the HCV replication status [96], and higher levels of plasma adiponectin may predict poor HCC survival in patients without liver transplantation [97]. However, serum adiponectin was found to be decreased in patients with HCC and to be inversely correlated with tumor size and number [98]. Moreover, in patients with HCV-related cirrhosis, serum adiponectin levels were significantly lower in patients who also had HCC, and the serum adiponectin level was significantly negatively correlated with both the overall tumor size and the number of tumor foci [99]. Lower serum total and HMW adiponectin levels were independent risk factors for the higher histological grade of HCC [100]. However, high serum levels of adiponectin were associated with higher all-cause, liver-unrelated, and liver-related mortality [101].

##### PAI-1

Although serum PAI-1 levels have been identified as positive predictors of the response to IFN-based therapy in G1 HCV-infected patients [102], another study of G1, G2, G3, and G6 HCV-infected patients showed no difference in pretherapy PAI-1 levels between patients with and without SVR. The study also demonstrated that the PAI-1-rs-1799889 and IFN-λ3-rs12979860 genotypes longitudinally affect the PAI-1 level and that patients with SVR showed increasing PAI-1 levels with escalating cardiovascular risk [103].

##### Visfatin

The serum visfatin concentration was found to increase significantly in patients with chronic HCV infection compared with controls [104,105] and was closely related to the low-density lipoprotein cholesterol level [106] and fibrosis score [107]. In patients with different stages of HCV infection, the plasma visfatin level was associated with the presence of HCC [108]. Consistent with this finding, the serum levels of visfatin differed significantly among HCC, HCV, and normal control groups, and the visfatin level was associated with liver cirrhosis in HCV-infected patients [109]. On the other hand, no correlation between visfatin and HCV genotype, viral load, or treatment response to IFN-based therapy has been shown [107].

##### RBP4

In a cross-sectional study, patients with chronic HCV infection had lower RBP4 levels than did control subjects, and higher RBP4 levels were linked to lower ALT levels, hyperlipidemia, and high HOMA-IR scores [110]. Moreover, a significant decrease in serum RBP4 levels in patients with advanced stages of disease due to HCV infection was reported [111]. Consistent with this finding, an inverse association between the serum RBP4 concentration and the fibrosis stage was found in patients with HCV infection [112]. However, in the JFH1 infectious cell culture system, HCV core protein-enhanced RBP4 levels, and partial knockdown of RBP4 had a positive impact on HCV replication [113]. Only patients with SVR after IFN-based therapy had higher RBP4 levels post-therapy than at baseline [114].

##### Resistin

Hyperresistinemia in patients with chronic HCV infection has been consistently reported [115,116,117,118,119]. This condition is reversed after viral clearance [55,120,121] and determines moderate to severe fibrosis [117]. Our previous study showed that resistin originates primarily from intrahepatic lymphocytes, stellate cells, Kupffer cells, hepatic progenitor cells, and hepatocytes in HCV-infected patients [120]. Although the baseline resistin level was reported to be unassociated with therapeutic response [55], fine-tuned by resisin SNPs including RETN-rs34861192, RETN-rs3219175, RETN-rs3745367, and RETN-rs1423096, the intrahepatic, multicellular resistin reinforced IFNL3 in eliminating HCV via immunomodulation [120]. Moreover, high serum resistin levels might allow early identification of patients with cirrhosis who are at substantially increased risk of HCC [121].

##### Chemerin

Serum chemerin levels were significantly higher in patients with HCV infection than in controls, although chemerin levels were negatively associated with the necroinflammatory stage [122]. On the other hand, there was a negative association between serum chemerin and hepatic chemerin expression, which was not associated with necroinflammatory activity, steatosis grade, fibrosis stage, or metabolic abnormalities in HCV-infected patients [123].

##### Multiple Adipokines

Adiponectin, leptin, and visfatin have been found to be associated with liver cirrhosis in HCV-infected patients [109]. Sex was associated with leptin and adiponectin levels, and body mass index (BMI) was associated with leptin and PAI-1 levels in HCV-infected patients at baseline. Among patients achieving SVR, at 24 weeks post-IFN-based therapy, sex and BMI were associated with leptin, adiponectin, and PAI-1 levels; hepatic steatosis and the aspartate aminotransferase-to-platelet ratio index with adiponectin levels; and the HOMA-IR score and HCV genotype with PAI-1 levels [62]. Serum leptin levels were higher in G1 HCV-infected patients than in G3 HCV-infected patients, and serum resistin levels were higher in G3 HCV-infected patients [116]. In patients with compensated HCV-associated cirrhosis, insulin resistance but not the serum levels of adiponectin and leptin predicted the occurrence of HCC and of liver-related death or transplantation [124]. The serum levels of leptin and resistin and the leptin-to-adiponectin ratio were significantly higher in patients with chronic HCV infection than in controls, and low serum levels of resistin were associated with the presence of fibrosis independent of potential confounders [115]. In nonobese HCV core transgenic mice, hepatic steatosis is associated with downregulated leptin gene and hypoadiponectinemia, and these effects may be ameliorated by adiponectin treatment [91].

### 2.3. Autoimmune Liver Disease

#### 2.3.1. Primary Biliary Cholangitis (PBC)

PBC predominantly affects middle-aged women and is a rare, chronic progressive cholestatic liver disease characterized by the autoimmune-mediated destruction of the small- and medium-sized intrahepatic bile ducts [125].

##### Leptin

Most studies of adipokines in PBC patients involving leptin have shown diverse results. Leptin levels have been reported to be either higher [126,127] or lower [128,129,130] in PBC patients than in controls. Leptin levels have been reported either to be associated with the histological stage of PBC [130] or to be unrelated to disease severity [128].

##### Adiponectin and Resistin

Adiponectin and resistin levels have been reported to be higher in PBC patients than in controls [127].

#### 2.3.2. Autoimmune Hepatitis (AIH)

AIH is a rare, immune-mediated, inflammatory condition of the liver that is characterized by circulating autoantibodies, hypergammaglobulinaemia, and distinctive features on liver biopsy [131].

##### Adiponectin

A positive association between inflammation and adiponectin is usually reported in inflammatory/immune pathologies, in contrast with the negative correlation typical in metabolic diseases [132]. For example, patients with AIH showed significantly higher adiponectin concentrations than controls despite their higher HOMA-IR scores [133].

### 2.4. Alcoholic Liver Disease (ALD)

ALD, caused by excess and chronic alcohol intake [134], is a complex disorder with a disease spectrum ranging from steatosis to steatohepatitis, cirrhosis, and HCC [135]. Alcohol is primarily metabolized in the body via diverse pathways by the catalytic activity of three different enzymes–alcohol dehydrogenase, cytochrome P450 2E1 (CYP2E1), and catalase. Studies on alcoholic patients and rodent models have shown that chronic ethanol consumption reduces adipose tissue mass and causes CYP2E1-mediated oxidative stress and inflammation of adipose tissue [134].

#### 2.4.1. Leptin

The effects of alcohol on circulating leptin are not consistent and may be related to changes in fat mass instead of the alcohol per se. Leptin has been reported to be increased, decreased, or unchanged across a range of rodent models of chronic alcohol administration. Likewise, the serum leptin levels from humans appear to be unrelated to alcohol intake, although exceptions do exist. In alcoholic patients, leptin levels have been reported to be increased, decreased, or unchanged, and serum leptin levels were not altered by either alcohol withdrawal or the severity of liver disease [136]. However, in alcohol-dependent patients with cirrhosis, leptin is significantly higher before liver transplantation and decreases significantly after transplantation. Moreover, alcohol-dependent patients on the waiting list had significantly higher leptin promoter methylation values than patients who underwent liver transplantation for other reasons [137].

#### 2.4.2. Adiponectin

Alcohol exhibits a specific effect on serum adiponectin levels that is dose- and time-dependent and is correlated with the degree of hepatic damage. Moreover, alcohol does not seem to affect adiponectin expression in adipocytes directly but potentially affects it via mediators systemically released as a result of chronic alcohol intake [138]. The majority of data garnered from animal models of chronic alcohol consumption show circulating adiponectin levels to be decreased, although a few report no change. Conversely, serum adiponectin levels in humans were increased in relation to alcohol consumption, although two investigations did report a dose-dependent decrease [136]. In addition, a study of cirrhosis and control patients showed that transplant-free survival was significantly lower among patients with alcoholic liver disease and adiponectin ≥17 μg/mL. Adiponectin levels were associated with the intensity of liver dysfunction and worse prognosis in patients with alcoholic liver disease, suggesting its potential as a prognostic biomarker [139]. Emerging evidence has revealed that dysregulated adiponectin-fibroblast growth factor (FGF) 15 (human homolog, FGF19) axis and impaired hepatic adiponectin-FGF15/19 signaling are associated with alcoholic liver damage in rodents and humans [140].

#### 2.4.3. Other Adipokines

The levels of chemerin decrease with the progression of liver damage during alcoholic liver cirrhosis [141]. In addition, a novel adipose tissue-derived cytokine, C1q TNF-related protein-3 (CTRP3), was shown to attenuate hepatic triglyceride accumulation in response to long-term chronic, but not short-term, alcohol consumption [142].

## 3. Adipokines and the Pancreas

### 3.1. Pancreatic Cancer

Human pancreatic adipocytes store lipids and release adipokines in response to the overall metabolic, humoral, and neuronal status [143]. Fatty pancreas is associated with age, BMI, and diabetes, which are risk factors for pancreatic cancer [144]. In particular, expansion and inflammation of visceral adipose tissue induce insulin resistance that fosters systemic secretion of insulin and insulin-like growth factor 1 [145].

#### 3.1.1. Leptin

Elevated leptin may promote pancreatic tumor invasion and metastasis, activating the Janus kinase 2 (JAK2)/signal transducer and activator of transcription 3 (STAT3) axis [145].

#### 3.1.2. Adiponectin

Reduced release of adiponectin was found to decrease the tumor-suppressive effects of adiponectin in a manner mediated by JAK2/STAT3 inhibition and downregulation of intracellular β-catenin [145].

### 3.2. Insulin Resistance and Diabetes

Insulin resistance is characterized by a diminished response to insulin stimulation, resulting in the failure of target tissues to adequately dispose of blood glucose, inhibit lipolysis, stimulate glycogen synthesis, and inhibit hepatic glucose output and is a precursor event to type 2 diabetes [146].

#### 3.2.1. Leptin

Adipokines preferentially affect islet vasculature [147]. Pancreatic hormones play a role in energy balance, exerting short-acting control, while insulin and leptin derived from adipose tissue are involved in long-acting adiposity signaling and regulate body weight [148]. Leptin receptors are widely expressed in peripheral tissues, including the beta (β) cells of the endocrine pancreas [149], and their activation directly inhibits insulin secretion from these endocrine cells. Additionally, β cell mass can be affected by leptin through changes in proliferation, apoptosis, or cell size [150]. Specifically, insulin is adipogenic, increases body adipose tissue mass, and stimulates the production and secretion of leptin, which acts centrally to reduce food intake and increase energy expenditure. Leptin, in turn, suppresses insulin secretion by both central actions and direct actions on β cells. Because leptin levels are directly proportional to body adipose tissue mass, an increase in adiposity increases plasma leptin, thereby curtailing insulin production and further increasing fat mass [151], thus establishing a hormonal regulatory feedback loop, the adipo-insular axis [151]. In addition, leptin exerts a tonic inhibitory effect on β cell excitability via its ability to increase the plasma membrane ATP-sensitive K+ (KATP) channel density and whole-cell KATP channel current [152]. In most overweight individuals, physiological regulation of body weight by leptin is likely disturbed, constituting leptin resistance. This leptin resistance at the pancreatic β cell level may contribute to dysregulation of the adipo-insular axis and accelerate the development of hyperinsulinemia and can manifest as diabetes mellitus in overweight patients [153]. On the other hand, leptin might be used as an adjunct to insulin therapy in patients with insulin-deficient diabetes, providing insight into its therapeutic properties as an antidiabetic agent [154]; moreover, leptin monotherapy has been reported to reverse type 1 diabetes independent of insulin [155]. Because ob/ob mice lack functional leptin, they develop severe insulin resistance with hyperglycemia and hyperinsulinemia and are described as a model for the prediabetic state. Although ob/ob mice have large pancreatic islets, their β cells respond adequately to most stimuli [156].

#### 3.2.2. Other Adipokines

In addition to leptin, other adipokines, including adiponectin and visfatin (i.e., eNAMPT), apelin, resistin, RBP4, fibroblast growth factor 21, nesfatin-1, and fatty acid binding protein 4 directly regulate β cell function [157,158]. In particular, adiponectin has received considerable attention for its potential antidiabetic actions. By stimulating adipogenesis, opposing inflammation, and influencing rates of lipid oxidation and lipolysis, adiponectin critically governs lipid spillover into nonadipose tissues [159]. Moreover, adiponectin stimulates insulin secretion and has antiapoptotic properties in β cells [160]. Resistin antagonizes insulin action, and it is downregulated by rosiglitazone and peroxisome proliferator-activated receptor gamma agonists [161]. Interestingly, visfatin does not exert insulin-mimetic effects in vitro or in vivo but rather exhibits robust nicotinamide adenine dinucleotide (NAD) biosynthetic activity. NAMPT-mediated systemic NAD biosynthesis is critical for beta cell function, suggesting a vital framework for the regulation of glucose homeostasis [162].

## 4. Adipokines and the Alimentary Tract

### 4.1. Esophagus

Almost all cases of esophageal adenocarcinoma arise from underlying Barrett’s esophagus, a metaplastic change in the esophagus [163]. Moreover, central obesity is involved in the pathogenesis and progression of Barrett’s esophagus to esophageal adenocarcinoma [164,165] and GERD, a disorder due to the retrograde flow of refluxate into the esophagus [166]. Barrett’s esophagus, esophageal adenocarcinoma, and GERD thus might be associated with adipokine alterations.

#### 4.1.1. Leptin

In obese patients with GERD, leptin, and ObR levels were found to be higher and lower, respectively, than in nonobese patients with GERD [167,168]. Consistent with this finding, leptin resistance in individuals with overweight and obesity is associated with features of GERD, and leptin levels are positively associated with frequent GERD symptoms [169] and with the clinical and endoscopic severity of GERD [170]. The multi-biomarker score derived from multiple parameters, including leptin levels and GERD frequency and duration, can identify patients with Barrett’s esophagus [171]. Moreover, leptin levels were found to be positively associated with Barrett’s esophagus; this association was stronger in men with GERD than in women with GERD [172], and serum leptin levels might be associated with an increased risk of Barrett’s esophagus among men but not women [165]. Through enhancing macrophage migration inhibitory factor-induced inflammatory signaling, leptin may contribute to the development of GERD [173]. In addition, leptin stimulates cell proliferation and inhibits apoptosis in OAC cells via extracellular signal-regulated kinase (ERK), p38 mitogen-activated protein kinase (MAPK), phosphoinositide 3-kinase (PI3K)/Akt, and JAK2-dependent activation of cyclooxygenase-2 (COX-2) and prostaglandin E2 (PGE2) production [174]. Leptin receptors are highly expressed on esophageal epithelial cells. The finding that patients with Barrett’s esophagus had higher fundic leptin levels than individuals with a normal esophagus indicates that ObR expression on esophageal epithelial cells provides a pathway for leptin-mediated signal transduction [175]. In particular, the oncogenic effect of leptin has been reported to modulate the cellular response to radiation [176], angiogenesis and lymphangiogenesis [177], and chemoresistance in gastroesophageal adenocarcinomas [178], as well as to stimulate the proliferation, invasion, and migration and inhibit the apoptosis of OE33 esophageal adenocarcinoma cells [179].

#### 4.1.2. Adiponectin

The anti-inflammatory effects of adiponectin are specific to its individual multimers, with low-molecular-weight (LMW) adiponectin being the most anti-inflammatory. High levels of LMW adiponectin are associated with a decreased risk of Barrett’s esophagus among patients with GERD [180]. Consistent with this finding, serum adiponectin was found to be inversely associated with Barrett’s esophagus, particularly in men [181]; in patients with GERD, erosive esophagitis and Barrett’s esophagus were found to be associated with decreased adiponectin levels compared to those in patients without GERD [182]; and low serum adiponectin levels may be associated with an increased risk for erosive esophagitis [183]. However, in a study of 863 cases, adiponectin levels were positively associated with the risk of Barrett’s esophagus in patients with GERD and in smokers but not in a control population without GERD symptoms [184].

#### 4.1.3. Leptin and Adiponectin

A systematic review showed that increased serum levels of leptin are associated with an increased risk of Barrett’s esophagus. In contrast, increased total serum levels of adiponectin do not seem to modify the risk of Barrett’s esophagus [185]. Similarly, the adjusted odds ratios for Barrett’s esophagus were 8.02 for the highest quintile vs. the lowest quintile of leptin level, while there were no differences in adiponectin levels between the cases and controls [186]. An increased level of leptin was associated with an increased risk for esophageal adenocarcinoma, whereas an increased level of HMW adiponectin was inversely associated with esophageal adenocarcinoma [187]. Interestingly, higher adiponectin levels were found in patients with esophageal squamous cell carcinoma (SCC) than in patients with esophageal adenocarcinoma [188], and resistin may be a biomarker for esophageal SCC [189].

### 4.2. Stomach

#### Leptin

Similar to adipose tissue, the stomach simultaneously expresses leptin and ObR. Leptin maintains energy homeostasis with the aid of its antagonistic hormone ghrelin [190]. Ghrelin is a gut-derived peptide hormone that was first isolated from the stomach [191]. Ghrelin stimulates appetite and controls gastric motility and acid secretion [192]. Collectively, leptin and ghrelin are known as “hunger hormones”. In addition, leptin signaling can affect the gastric mucosal milieu [193]. Adipose tissue secretes leptin in a slow constitutive endocrine manner, and the gastric mucosa releases leptin in a rapidly regulated exocrine manner into the gastric juice. Thus, adipocytes and gastric epithelial cells are two cell types in which metabolism is closely linked to food intake and energy storage [194]. Moreover, overexpression of leptin and phosphorylated ObR is implicated in gastric cancer, and diet-induced obesity causes precancerous lesions in the mouse stomach [193].

### 4.3. Small Intestine

#### Leptin

Creeping fat, characterized by hyperplasia of the mesenteric fat, which creeps around inflamed segments of the small intestine [195], can be distinguished from healthy adipose tissue by its distinctively small adipocytes with high levels of adipokines and dominant immune cell infiltration. In particular, leptin has been reported to enhance the maturation of the systemic and intestinal immune systems in preterm conditions [196].

### 4.4. Colon

#### 4.4.1. Colitis

Inflammatory bowel diseases (IBDs) comprise chronic inflammatory disorders of the gastrointestinal tract, affecting millions worldwide [197]. The exact etiopathogenesis of IBD remains unknown, while potential factors involve genetic predisposition, environmental conditions, and immunological dysfunctions. The main IBDs are ulcerative colitis (UC) and Crohn’s disease (CD) [198]. Although transmural inflammation in CD may affect any part of the gastrointestinal tract, it occurs most frequently in the terminal ileum or the large intestine. In contrast, UC usually occurs only in the large intestine and is limited to the mucosal layer [199]. Obesity-induced chronic inflammation increases the risk of UC and CD [200]. Mesenteric adipose tissue (MAT) hyperplasia is a hallmark of CD. Mesenteric adipose-derived stromal cells (ADSCs) synthesize and release adipokines in a disease-dependent manner and alter colonic epithelial cell signaling [201].Transmural inflammation facilitates bacterial translocation into the creeping fat, which exerts a protective effect via a localized anti-inflammatory effect, thus preventing a systemic inflammatory response in CD [202].

##### Leptin

Leptin may regulate dendritic cell migration from the gut under homeostatic and inflammatory conditions, linking mesenteric obesity and inflammation in CD [203]. However, activation of ObR is an important pathogenic mechanism of UC, and ObR deficiency may confer resistance to 2,4,6-trinitrobenzene sulfonic acid (TNBS)-induced colitis by inhibiting the nuclear factor κ-light-chain-enhancer of activated B cells (NF-κB) and Ras homolog gene family member A (RhoA) signaling pathways [204]. Moreover, luminal leptin is likely an intestinal chloride secretagogue, particularly when present at elevated concentrations or in the setting of inflammation [205]. Intraperitoneal administration of leptin to lean rats increased colonic epithelial permeability and altered zonula occludens-1 expression and organization [206], and the increased mucosal leptin may interact with mast cells and the nervous system to enhance diarrhea-predominant irritable bowel syndrome [207]. On the other hand, the protective mucosal immune function of leptin in *Clostridium difficile* colitis is partially mediated by a leptin-STAT3 inflammatory pathway that is defective as a result of the ObR Q223R mutation [208].

##### Adiponectin

In contrast to the proinflammatory role of leptin, adiponectin maintains intestinal homeostasis and protects against murine colitis through interactions with its receptor AdipoR1 and by modulating adaptive immunity [209]. For example, adiponectin injection alleviated colonic injury and rectal bleeding in mice, downregulated colonic interleukin 1β (IL-1β), and TNF-α expression, and regulated apoptosis-related gene expression to attenuate dextran sodium sulfate (DSS)-induced colonic inflammation [210,211]. In addition, adiponectin markedly reduced the serum lipopolysaccharide concentration, a biomarker for intestinal integrity, and enhanced colonic expression of tight junction proteins [211]. Adiponectin expression was significantly suppressed by induction of colitis [212], and intracolonic silencing of adipoR1 in mice exacerbated TNBS-induced colitis [213]. However, whether adiponectin aggravates [214] or attenuates [215] DSS-induced colitis in adiponectin knockout mice remains controversial.

##### Leptin and Adiponectin

Overall, colitis induces a decrease in the levels of the mRNAs encoding leptin and adiponectin in MAT but an increase in the levels of mRNAs encoding inflammatory markers. Specifically, MAT in patients with inflammatory bowel disease shows a loss of the adipose profile and a greatly enhanced inflammatory profile [216].

#### 4.4.2. Diverticulosis

Creeping fat can be observed in CD. Interestingly, adipose tissue also frequently covers the basolateral site of inflamed diverticula, thus locally reflecting the phenomenon seen in CD. This finding suggests that each inflamed diverticulum mechanistically reflects CD on a miniature scale [217].

##### Leptin and Adiponectin

Leptin levels were found to be positively associated with diverticulosis, and LMW adiponectin levels were inversely related to the presence of diverticulosis in asymptomatic men [218].

#### 4.4.3. Colon Polyps and Cancer

Colorectal cancer (CRC) is the third most common cancer in men and the second most common in women worldwide. Most CRCs arise from colonic polyps, particularly adenomatous polyps [219]. The polyp size, number, and pathological findings are crucial prognostic factors for CRC. Nonadvanced colonic polyps are defined as one to two adenomatous polyps each <10 mm in size, and advanced colonic polyps are defined as any adenomatous polyp ≥10 mm in size or with >25% villous histology or high-grade dysplasia [220]. Obesity is a risk factor for both adenomatous polyps and CRC development [219], which likely results in adipokine alteration.

##### Leptin

In asymptomatic men, serum leptin levels were found to be significantly associated with the presence of tubular adenoma [221]. Leptin expression was more frequently observed in colon adenomas, especially in larger adenomas and adenocarcinoma in situ, than in normal colon tissues, but blood leptin levels were not found to be related to tissue leptin expression [222]. Tissue microarray analysis showed that leptin was gradually expressed during the normal-adenoma-adenocarcinoma sequence, suggesting an association between leptin and colorectal carcinogenesis. Intriguingly, high leptin expression was an indicator of favorable tumor features and better survival in CRC patients [223]. ObR is overexpressed in CRC cells, which may influence patient outcomes [224]. Both leptin [225] and ObR [225,226] were found to be present at higher levels in cancerous tissues than in adjacent colon tissues. Moreover, high circulating levels of ObR were found in patients with advanced-stage colon cancer [227]. However, a study of 2258 cases showed that soluble ObR levels were strongly inversely associated with CRC, whereas leptin was not associated with the risk of CRC [228]. Moreover, ObR was significantly correlated with early-stage and well-differentiated primary CRCs [229]. ObR expression was found to be higher in CRCs than in the corresponding normal mucosa, and ObR expression in tumors might be involved in the adaptive immune response in sporadic CRCs, likely via a microsatellite instability-high phenotype orientation [230]. Intriguingly, patients with ObR-positive tumors were found to have significantly better overall survival than those with ObR-negative tumors, and Ob-R is a prognostic marker associated with more favorable survival [229]. In human colon cancer, upregulation of leptin pathway members was found, and a large network of dysregulated transcripts was linked to poorer overall survival [231]. For example, leptin might regulate the proliferation, apoptosis, or invasion of CRC cells through the PI3K/Akt/mammalian target of rapamycin (mTOR) [232,233], nuclear factor erythroid 2-related factor 2 (Nrf2)-dependent Silent Information Regulator 2 Homolog 1 (SIRT1) [234], ERK1/2 [235,236,237], MAPK [236,237,238], JAK2, STAT3, activator protein 1 (AP-1) [239,240] and NF-κB [241] signaling pathways. In addition, leptin regulates proinflammatory genes such as interleukin 6 (IL-6), IL1β, and chemokine (C-X-C motif) ligand 1 (CXCL1) [242], and induces preneoplastic colon epithelial cells to orchestrate vascular endothelial growth factor (VEGF)-driven angiogenesis and vascular development [243]. In leptin-deficient ob/ob and ObR-deficient db/db mice, colon tumor growth was inhibited, although the animals exhibited severe obesity [226]; moreover, in leptin-deficient ob/ob mice, the presence of abnormally dense mucus-filled goblet cells suggested the possible involvement of leptin in tissue injury and/or mucosal defense mechanisms. Furthermore, in human colonic goblet-like HT29-MTX cells expressing ObR, leptin increased mucin secretion by activating protein kinase C (PKC)- and PI3K-dependent pathways [244].

##### Adiponectin

In contrast to leptin, adiponectin protects against chronic inflammation-induced colon cancer (CICC) [245] and demonstrates beneficial effects on colon cancer [209]. Adiponectin may be involved in reducing the severity of CICC by preventing goblet cell apoptosis and increasing epithelial-to-goblet cell differentiation [246]. Plasma adiponectin levels have been found to be inversely associated with colonic polyps, multiple colonic polyps, high-risk colonic polyps [247], early-stage CRC [248,249], and CRC stage [249]. Consistent with these findings, adiponectin negatively regulates colorectal cell survival and migration [250]. Both adiponectin and AdipoRon, a small molecule adiponectin receptor agonist, were found to suppress colon cancer risk in part by reducing the number of leucine-rich repeat-containing G protein-coupled receptor 5+ (Lgr5+) stem cells in mouse colonic organoids [251]. In a study of 2412 cases, non-HMW but not HMW adiponectin was associated with CRC risk [252]. The expression of AdipoR1 has consistently been reported to be higher in cancerous than in normal colonic tissues [253,254], while the expression of AdipoR2 has been reported to be lower [255] or higher [256] in cancerous tissues. Low plasma adiponectin levels were found to be associated with KRAS-mutant CRC risk but not with KRAS wild-type cancer risk [256]. Additionally, adiponectin might inhibit the growth of colon cancer cells by stimulating AMPK activity [257,258], thereby downregulating the mTOR pathway [259]. Additionally, adiponectin might regulate IL1β-induced colon carcinogenesis [260]. On the other hand, adiponectin signaling plays a role in modulating cellular cholesterol homeostasis, plasma membrane biophysical properties, and Wnt-driven signaling [261]. Adiponectin treatment suppresses angiogenesis in colon cancers. In vitro studies showed that adiponectin directly controls the malignant potential (cell proliferation, adhesion, invasion, and colony formation) and regulates metabolic (AMPK/70-kDa ribosomal protein S6), inflammatory (STAT3/VEGF), and cell cycle (p21/p27/p53/cyclins) signaling pathways in a liver kinase B1 (LKB)-dependent manner [261]. However, in another study, adiponectin levels were not correlated with visceral fat in the CRC and adenoma groups [262]. The responsiveness of colonic stem cells to adiponectin in diet-induced obesity is impaired and may contribute to the accumulation of stem cells observed in obesity [263]. Moreover, adiponectin was found to suppress colonic epithelial proliferation via inhibition of the mTOR pathway under high-fat diet but not basal diet feeding conditions [264]. A significant inverse correlation was found between the number of dysplastic aberrant crypt foci (ACF) and the plasma adiponectin level. Consistent with this finding, enhanced formation of ACF and tumors was observed in adiponectin-deficient mice [265], whichdevelop more intestinal tumors than wild-type mice [258], and adiponectin administration suppressed the growth of implanted tumors, causing larger central necrotic areas in the mice [261]. However, elevated levels of circulating adiponectin in adiponectin transgenic mice did not confer protection against colon tumor development [266]. The rs12733285C/T genotype and the A allele of rs1342387 (A/G or A/A) of ADIPOR1 are protective factors for CRC, while the rs266729G/C genotype and the G allele of ADIPOQ are risk factors for colon cancer [267]. Together, given that the concentration of adiponectin is high in serum, these findings indicate that the main role of adiponectin is likely homeostasis regulation rather than action as an anticancer adipokine. However, as the above epidemiological evidence shows, a low adiponectin level may be a basic risk factor for CRC. It is likely that the colonic epithelium is stimulated by specific carcinogens and that cancer development is then facilitated underhypoadiponectinemia [268].

##### Leptin and Adiponectin

Serum leptin and AdipoR1 and AdipoR2 expression levels were found to be associated with lymph node involvement, and AdipoR1 expression was correlated with tumor size in colon cancer patients [269]. Ionizing radiation can persistently decrease the levels of AdipoR1 and AdipoR2 but increase those of leptin and ObR and activate downstream proliferative pathways, for example, upregulating PI3K/Akt and JAK2 signaling, which may contribute to carcinogenesis [270]. Regarding sexual dimorphism, plasma adiponectin levels were found to be associated with a reduced risk of CRC among men but not among women [271].

##### Other Adipokines

Serum resistin levels in patients with colon cancer are elevated and correlated with tumor grade. Resistin binds to Toll-like receptor 4 (TLR4) on the colon cancer cell membrane and initiates TLR4-myeloid differentiation primary response 88 (MYD88)-dependent activation of ERK [272,273]. In addition, the resistin C-420G and G+299A polymorphisms have potential roles in the genetic predisposition to colon cancer [274]. High serum levels of YKL-40 (also called Chitinase 3-like 1) are associated with CRC in subjects without comorbidities [275] and are correlated with poor prognosis in patients with colon cancer [276]. Colon adenoma risk is associated with high circulating levels of RBP4 [277]. Finally, chemerin is thought to exert chemotactic, adipogenic, and angiogenic functions. Higher chemerin levels are associated with CRC risks [278,279]

**Figure 2:**
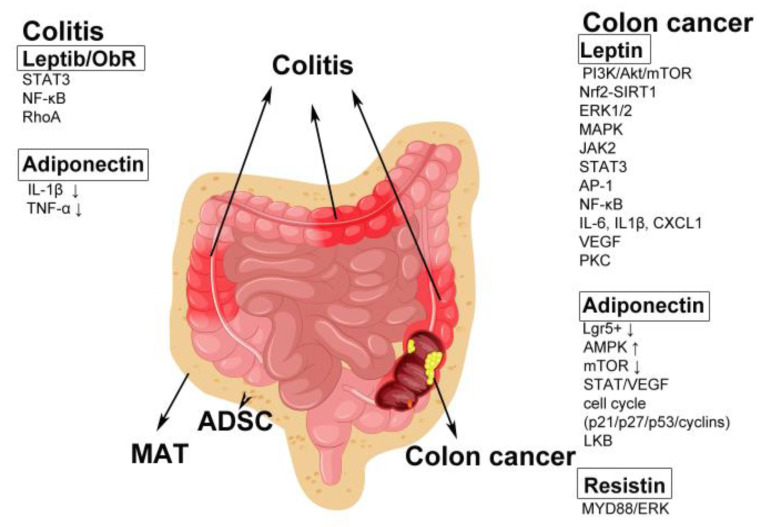
The adipokine-associated signaling pathways in colitis and colon cancer. The altered adipokine and associated pathways are shown on the left for colitis and on the right for colon cancer. Up arrows: upregulation of the signaling pathways under the stimulation of associated adipokines; down arrows: downregulation of the signaling pathways under the stimulation of associated adipokines. MAT: mesenteric adipose tissue; ADSC: adipose tissue-derived stem cells; ObR: leptin receptor; STAT3: signal transducer and activator of transcription 3; NF-êB: nuclear factor ê-light-chain-enhancer of activated B cells; RhoA: Ras homolog gene family, member A; IL-1â: interleukin 1â; TNF-á: tumor necrosis factor-á; PI3K: phosphatidylinositol 3-kinase; mTOR: mammalian target of rapamycin; Nrf2: nuclear factor erythroid 2-related factor 2; SIRT1:silent information regulator 2 homologue 1; ERK1/2: extracellular signal-related kinase 1/2; MAPK: mitogen-activated protein kinase; JAK2: Janus kinase 2; AP-1: activator protein 1; IL-6: interleukin 6; CXCL1: chemokine (C-X-C motif) ligand 1; VEGF: vascular endothelial growth factor; PKC: protein kinase C;Lgr5+: leucine-rich repeat-containing G-protein coupled receptor 5+; AMPK: MP-activated protein kinase; LKB: liver kinase B1; MYD88: myeloid differentiation primary response 88.

A schematic summarizing colon disease-associated adipokine alterations and the basis is provided in Figure 2.

## 5. Adipokines and the Biliary Tract

### 5.1. Leptin

Although higher leptin concentrations in the hepatic vein were found in bile duct ligated-(BDL) rats than in lean sham-operated rats, and colocalization of leptin and α-smooth muscle actin in activated hepatic stellate cells (HSCs) was observed by immunohistochemistry [280], the TNF-α-associated upregulation of leptin in dimethylnitrosamine (DMN)-induced but not in BDL-induced cirrhotic rats is consistent with a difference in the roles of TNF-α in rats with nonbiliary cirrhosis and those with biliary cirrhosis [281]. Regarding cholangiocarcinoma, leptin was found to increase the proliferation and metastatic potential of cholangiocarcinoma cells through STAT3-dependent activation of ERK 1/2. Moreover, loss of leptin function suppressed the development of cholangiocarcinoma [282]. Consistent with this finding, leptin increased the epithelial-mesenchymal transition and proangiogenic capability of cholangiocarcinoma cells, inhibited endogenous miR-122 expression, and upregulated pyruvate kinase muscle isozyme M2 [283].

### 5.2. Adiponectin

High levels of adiponectin were found in BDL rats [284], reflecting the antifibrotic role of adiponectin, as adiponectin overexpression in activated HSCs was found to reduce the proliferation but augment the apoptosis of HSCs [280]. Consistent with this finding, adiponectin protected the rat bile duct against early warm ischemia-reperfusion injury by suppressing the inflammatory response and hepatocyte apoptosis and NF-κB (p65) played an important role in this process [285].

### 5.3. Resistin

Hyperinsulinemia might upregulate the resistin gene in BDL-related cirrhosis [286].

## 6. Adipokines and the Gallbladder

Obesity, diabetes, and hyperlipidemia are known risk factors for the development of gallstones [287], and there is convincing evidence that excess body weight is associated with an increased risk for gallbladder cancer [288]. Gallbladder diseases, therefore, potentially lead to adipokine alteration.

### 6.1. Leptin

Prepregnancy obesity and the serum leptin concentration are strong risk factors for pregnancy-associated gallbladder disease [289], although a human study showed that the serum leptin concentration might not be a better predictor of gallbladder disease than anthropometry [290]. Leptin was found to promote cholesterol crystallization and gallstone formation [291] and, consistent with this finding, was reported to affect the components and secretion of bile in leptin-deficient mice. Furthermore, gallbladder diseases such as cholelithiasis are associated with serum leptin levels in humans [292] and dogs [293]. Leptin influences gallbladder bile volume, sodium, and pH, as well as numerous inflammatory cytokine genes and genes related to water, sodium, chloride, and bicarbonate transport [294]. Obese leptin-deficient (ob-ob) mice have large gallbladder volumes with decreased contraction and are predisposed to gallstone formation [295,296], and administration of leptin to these mice causes weight loss and restores gallbladder function [295]. Both leptin and ObR are localized throughout the cytoplasm of luminal and glandular epithelial cells in the canine gallbladder [292] and in human gallbladder cancer tissues and cell lines [297]. ObR-deficient (db-db) obese mice have an increased gallbladder volume due to abnormal gallbladder motility [298], decreased biliary cholesterol saturation despite elevated serum cholesterol, and hepatic steatosis, and decreased cholesterol crystal formation [299]. A large body of evidence demonstrates that high BMI, as an approximation for general adiposity, is a risk factor for the development of gallbladder cancers [300]. Consistent with this observation, leptin was found to promote the proliferation, migration, and invasion of gallbladder cancer cells by increasing ObR expression through the SOCS3/JAK2/p-STAT3 signaling pathway [297].

### 6.2. Adiponectin

Hypoadiponectinemia has been reported to be associated with cholesterol gallstone formation in humans and to promote gallstone formation in mice [301].

A summary of adipokine alterations in various clinical digestive diseases is provided in Table 1. The alteration patterns might act as diagnostic markers or therapeutic targets for specific digestive diseases.

## 7. Conclusive Remarks and Future Challenges

Considering the current review, almost all digestive diseases are associated with altered adipokine profiles; with few exceptions, the unfavorable and favorable implications of leptin and adiponectin, respectively, have been consistently reported. However, gaps remain in understanding the precise roles of adipokines in digestive diseases. For example, in addition topatients with lipodystrophy and those with insulin-deficient diabetes, which patients will benefit from leptin therapy? Is adiponectin therapy a promising approach for most patients with digestive diseases? In addition, many associated mechanisms have been explored in vitro or in animal studies. Future prospective studies in largeindependent cohorts with identifiable outcomes for specific digestive diseases and sophisticated molecular investigations are required to verify the proposed basis and to investigate the therapeutic targets in confirming the fundamental mechanisms underlying the findings described herein.

## Figures and Tables

**Table 1:** Adipokine alterations in various digestive diseases *.

Diseases	Adipokines	Increased (I), Decreased (D), or No Changes (N)	Associated Findings (References)
NAFLD	Leptin	I	Increased severity [22]
	Adiponectin	D	Inversely related to the severity of steatosis [29], necroinflammation, and fibrosis [28]
	PAI-1	I	Independently associated with NAFLD [39]
Hepatitis B	Leptin	I/D	Associated with fibrosis/cirrhosis [44,45,46]/with cirrhosis/HCC [47]
	Adiponectin	I/D	Associated with viral load [48,49]/Viral load inversely associated with HDL-C [50]
	Resistin	I	Associated with hepatic necroinflammation [54]
	Visfatin	I	Negatively correlated with haptoglobin and fibrinogen [56]
Hepatitis C	Leptin	I/N	[65,66]/[67,68]
	Adiponectin	I/N/D	Associated with fibrosis [74,75,76,77,78,81] and inflammation [85]/[67]/in G1 and G3 HCV patients [84], associated with steatosis [79,84,86,87,88,89]
	Visfatin	I	[104,105]
	RBP4	D	Inversely associated with hepatic fibrosis [110,111,112]
	Resistin	I	Associated with hepatic fibrosis [115,116], reversed after viral clearance [55,120,121], associated with hepatic fibrosis [117]
	Chemerin	I	[122]
PBC	Leptin	I/D	[126,127]/[128,129,130]
	Adiponectin	I	[127]
	Resistin	I	[127]
ALD	Leptin	I, N or D	[136]
	Adiponectin	I or D	[136]
	Chemerin	I	[141]
Pancreatic cancer	Leptin	I	[145]
	Adiponectin	D	[145]
Diabetes	Leptin	I	[141]
GERD	Leptin	I	[167,168,173]
Barrett’s esophagus	Leptin	I	[171,185,186], stronger in men [166,172]
	Adiponectin	I/D/N	[184]/Among patients with GERD and among smokers [181], especially in patients with GERD [180,182]/[186]
Esophageal cancer	Leptin	I	Increased cellular response to radiation [176], angiogenesis and lymphangiogenesis [177], chemoresistance of gastro-oesophageal adenocarcinomas [178].
Colitis	Leptin	I	[203]
Diverticulosis	Leptin	I	[218]
	Adiponectin	D	[218]
Colon polyp	Leptin	I	Serum leptin associated with tubular adenoma [221], local leptin with colonic adenoma [222]
	Adiponectin	D	[247]
	RBP4	I	[277]
Colon cancer	Leptin	N	[227]
	Adiponectin	D	[248,249]
	Resistin	I	[262,263]
	YKL-40	I	In subjects without comorbidity [275] and correlated with poor prognosis in patients with colon cancers [276]
Cholelithiasis	Leptin	I	[289]

NAFLD: Non-alcoholic fatty liver disease; HCC: Hepatocellular carcinoma; HDL-C: high-density lipoprotein cholesterol; G1 and G3: genotype 1 and genotype 3; HCV: hepatitis C virus; PBC: primary biliary cholangitis; ALD: alcoholic liver disease; GERD: gastroesophageal reflux disease. *: data of in vivo or animal studies were not listed in the current table.

## Abbreviations

NAFLD	nonalcoholic fatty liver disease
PPAR-α	proliferator-activated receptor-alpha
ObR	leptin receptor
NASH	nonalcoholic steatohepatitis
AdipoR1	adiponectin receptor 1
TNF-α	tumor necrosis factor-alpha
BA	bile acid
SNP	single nucleotide polymorphism
PAI-1	plasminogen activator inhibitor-1
RBP-4	retinol-binding protein-4
HBV	hepatitis B virus
CHB	chronic hepatitis B
IFN-α	interferon-alpha
HCC	hepatocellular carcinoma
ALT	alanine aminotransferase
IFNL3	interferon λ3
DAA	direct-acting antiviral agent
G	genotype
SVR	sustained virologic response
C3	component 3
HMW	high-molecular-weight
PBC	primary biliary cholangitis
AIH	autoimmune hepatitis
BMI	body mass index
JAK2	Janus kinase 2
STAT3	signal transducer and activator of transcription 3
KATP	ATP-sensitive K+
GERD	gastroesophageal reflux disease
ERK	extracellular signal-regulated kinase
MAPK	mitogen-activated protein kinase
PI3K	phosphoinositide 3-kinase
COX-2	cyclooxygenase-2;
PGE2	prostaglandin E2
LMW	low-molecular-weight
SCC	squamous cell carcinoma
UC	ulcerative colitis
CD	Crohn’s disease
ADSC	adipose-derived stromal cells
TNBS	2,4,6-trinitrobenzene sulfonic acid
NF-κB	nuclear factor κ-light-chain-enhancer of activated B cells
RhoA	Ras homolog gene family, member A
IL-1β	interleukin 1β
DSS	dextran sodium sulfate
MAT	mesenteric adipose tissue
CRC	colorectal cancer
mTOR	mammalian target of rapamycin
Nrf2	nuclear factor erythroid 2-related factor 2
SIRT1	silent information regulator 2 homologue 1
AP-1	activator protein 1
IL-6	interleukin 6
CXCL1	chemokine (C-X-C motif) ligand 1
VEGF	vascular endothelial growth factor
PKC	protein kinase C
CICC	chronic inflammation-induced colon cancer
Lgr5+	leucine-rich repeat-containing G-protein coupled receptor 5+
AMPK	AMP-activated protein kinase
LKB	liver kinase B1
ACF	aberrant crypt foci
TLR4	Toll-like receptor 4
MYD88	myeloid differentiation primary response 88;
RETN	resistin
BDL	bile duct-ligated
DMN	dimethylnitrosamine
HSC	hepatic stellate cell
